# Grain Feeding Improves Yak Meat Tenderness and Lipid Deposition: Meat Quality, Amino Acid and Fatty Acid Profiles, Metabolomics, and Transcriptomics

**DOI:** 10.3390/foods15010172

**Published:** 2026-01-04

**Authors:** Bo Zou, Yuanli Yang, Yuqing Zhou, Yiran Yang, Weiru Song, Peng Xie, Mingwu Zang

**Affiliations:** 1 State Key Laboratory of Animal Nutrition and Feeding, Institute of Animal Sciences, Chinese Academy of Agricultural Sciences, Beijing 100193, China; bozoucau@hotmail.com (B.Z.);; 2 Haibei Comprehensive Experimental Station of National Beef Cattle & Yak Industrial Technology System, Haibei 810299, China; 3 Animal Disease Prevention and Control Centre of Yushu Tibetan Autonomous Prefecture, Yushu 815099, China

**Keywords:** meat quality, grain feeding, grass feeding, metabolomics, transcriptomics

## Abstract

Grain feeding is used to alleviate grazing pressure on the Tibetan Plateau. This study employed metabolomics and transcriptomics to elucidate the regulatory mechanisms of grain feeding on yak (Bos grunniens) meat quality, intramuscular fat, and amino acids. The results demonstrate that grain feeding significantly reduces meat shear force (11.05 vs. 18.98) and increases intramuscular fat content (1.48 g/100 g vs. 0.75 g/100 g). This is accompanied by elevated levels of monounsaturated and saturated fatty acids, alongside a decreased proportion of n-3 PUFAs, leading to a higher n-6/n-3 ratio of 5.13. Mechanistically, metabolomic analysis identified 83 differential metabolites, including flavor-related nucleosides, amino acids, and key lipids, such as palmitoleic and oleic acid, which collectively contribute to improved flavor and tenderness. Concurrently, transcriptomics revealed 1047 differentially expressed genes enriched in lipid metabolism pathways, including PPAR signaling, steroid biosynthesis, and glycerolipid metabolism. The PPAR signaling pathway plays a central role in coordinating lipid synthesis, and critical genes, such as PNPLA2, PPARA, SREBF1, and PRKAA1, were highlighted. In conclusion, grain feeding improves yak meat tenderness and fat deposition by modulating lipid metabolism at both the transcriptional and metabolic levels. This improvement, however, is balanced against a less favorable n-6/n-3 PUFA ratio.

## 1. Introduction

Relying on the natural grasslands of the plateau, a green livestock industry system based on ecological grazing has been formed. The yak, uniquely adapted to the Qinghai–Tibetan Plateau, is a vital resource that provides meat, milk, fiber, and manure to the population. Its meat, rich in protein and unsaturated fatty acids, holds significant market appeal [1]. However, year-round grazing on natural pastures, combined with harsh winters and scarce feed, limits weight gain in yaks [1]. To develop a high-quality and high-efficiency yak meat industry, researchers have attempted to address these issues by altering breeding methods [2,3].

The quality of animal meat is closely related to the way it is raised. For example, the tenderness attribute of beef is determined by three factors: background toughness, toughening phase, and tenderization [4]. Background toughness is the basis of beef tenderness, which is determined by the connective tissue component of muscle [5]. These muscle components will ultimately be influenced by the structure and content of fat and amino acids in beef. Muscle tissue with a higher fat content may reduce meat hardness [6]. Therefore, feeding regimens that alter fat and amino acid deposition can influence muscle toughness. Hu et al. [1] found that concentrated feed would increase the ratio of n-6/n-3 polyunsaturated fatty acids in yak meat, thereby changing the quality and nutritional characteristics of meat. Adding algae to high-energy feed can change the content of n-3 polyunsaturated fatty acids in mutton [2]. In addition, adding resveratrol to feed can enhance the tenderness of beef, reduce cooking loss, and maintain the stability of meat color [7].

Guo et al. [8] discovered, through metabolomics, that feeding with alfalfa leaves can improve pork quality by regulating lipid metabolism and antioxidant capacity. Also, the differences in meat quality and nutritional characteristics between Hu sheep and Durper sheep are related to fat synthesis [9]. Research has found that the impact of different feeding methods on beef quality is influenced by the expression of the FASN, FABP3, PLIN1, SLC16A13, FADS6, and SCD genes in the peroxisome proliferator-activated receptor (PPAR) signaling pathway [10]. However, the effects of different feeding methods on the meat quality and nutritional properties of plateau yaks and the underlying metabolic mechanisms remain insufficiently studied. Furthermore, studies that combine metabolomics and transcriptomics to explore the mechanisms underlying the effects of feeding methods on the quality and nutritional characteristics of yak meat are relatively rare.

This study compared the effects of two feeding methods, grass-fed and grain-fed groups, on the quality and nutritional characteristics of yak meat. The regulatory mechanisms of grain feeding on the quality, fat, and amino acids of yak meat were investigated using metabolomics and transcriptomics, providing a theoretical foundation for obtaining high-quality yak meat through optimized feeding methods.

## 2. Materials and Methods

### 2.1. Animal and Sampling

The animal experimental procedures were approved by the Chinese Academy of Agricultural Sciences. Before the experiment, all the yaks were managed uniformly, including their diet, feeding schedule, and feeding environment. A total of 12 healthy male yaks (2–3 years old, about 300 kg) with similar genetic backgrounds were randomly sampled in March from Qinghai Xiahua Halal Food Cooperation (Haibei, Qinghai, China) and divided into a grass-fed group (*n* = 6) and a grain-fed group (n = 6) randomly. In the grass-fed group, 6 yaks were only grazed in natural pastures with no supplements; in the grain-fed group, 6 yaks were fed with a total mixed ration (TMR) food in an enclosure for 6 months. All individuals were dewormed before the test and fed in Qinghai Province, China, where dominant vegetable types are kobresia humilis, leymus secalinus, elymus nutans, carex aridula, and potentilla acauli. The grass-fed yaks could freely eat grass with vegetables as the only feed. The grain-fed yaks were fed twice a day at 8:00 am and 8:00 pm every day. The health of animals was monitored by farmers every day. The diet formulation and calculated nutrient values are presented in Table S1. The main raw material composition contained VA, VD3, VE, nicotinamide, ferrous sulfate, alkaline copper chloride, zinc sulfate, manganese sulfate, calcium iodate, sodium selenite, cobalt chloride, calcium hydrogen phosphate, sodium chloride, calcium carbonate, propionic acid, rice shell powder, etc. The contents of amino acids and fatty acids in the total mixed ration are shown in Table S2. According to the Nutrient Requirements of Beef Cattle (NRC) (1994) recommendations for cattle, the TMR that satisfied the nutrient requirements of the growing stage was designed for yaks. By August, all individuals were slaughtered humanely, and the longissimus lumborum (LL) between the ribs of the carcasses was obtained for measurement.

### 2.2. Cooking Loss and Shear Force

Beef samples were put into a retort pouch and cooked in an 80 °C water bath until the central temperature reached 70 °C. The cooked samples were chilled to room temperature and reweighed. Cooking loss was calculated as a percentage of weight loss before and after cooking.

After determining cooking loss, the shear force of the meat samples was determined via a texture analyzer (Stable Micro Systems, Surrey, UK). A 3.0 × 1.0 × 1.0 cm^3^ meat stick was cut, and the shear force was measured. The results were in kg/cm^2^.

### 2.3. Intramuscular Fat and Protein

The crude fat content of the meat samples was determined via Soxhlet extraction. An approximately 5.0 g sample with the surface fat removed was weighed and dried to constant weight. The fat was extracted using a Soxhlet extractor (FOSS, Hilleroed, Denmark). The program was set to petroleum ether extraction at the boiling point of the solvent for 30 min, petroleum ether reflux for 60 min, and petroleum ether recovery for 20 min. The collection bottle with extract was dried to constant weight, and the crude fat content was then calculated.

The protein content was determined via spectrophotometry. A 0.5 g sample was digested with 0.1 g CuSO_4_, 1 g K_2_SO_4,_ and 5 mL H_2_SO_4_ in a 100 mL Kjeldahl flask. After that, the content in the flask was transferred to a 100 mL volumetric flask and diluted with water. Then, 5 mL of the above solution was added to another volumetric flask with a drop of p-nitrophenol indicator solution and shaken. NaOH and acetic acid were added successively to the volumetric flask with the digestion solution until the solution turned yellow and colorless, respectively. The volume of the volumetric flask was added to 100 mL. Then, 2 mL of the above-diluted solution, 4 mL of sodium acetate–acetic acid buffer, and 4 mL of a chromogenic agent were added to a 10 mL colorimetric tube and heated in a 100 °C water bath for 15 min. After cooling, the OD values (400 nm) were then measured using a UV/VIS spectrophotometer (UV-6000PC, Metash Instruments Co., Ltd., Shanghai, China).

### 2.4. Fatty Acid Profiles

Each sample was hydrolyzed by HCl and then saponified and methylated with 2% methanolic NaOH, 15% methanolic BF_3_, heptane, NaCl, and Na_2_SO_4_. Fatty acid methyl esters (FAMEs) were prepared with isooctane and NaHSO_4_. The triglycerides undecanonate was the internal standard. The obtained FAMEs were analyzed by gas chromatography (HP6890, Agilent Technologies Co., Ltd., Waldbronn, Germany) equipped with a flame-ionization detector and a polycyanopropyl siloxane column (100 m × 0.25 mm i.d. with 0.2 μm film thickness). Nitrogen gas was used as the carrier gas. The temperature of the FID was 280 °C.

### 2.5. Amino Acid Profiles

The amino acid content was determined by the method of Guo et al. [11] with slight modifications. The sample was hydrolyzed by 6 M HCl at 110 ± 1 °C for 22 h and concentrated by a vacuum concentrator (Taicang Huamei Biochemical Instrument Co., Ltd., Suzhou, Jiangsu, China). The sample was resolved by citrate sodium and determined by an amino acid autoanalyzer (Hitachi Co., Ltd., Tokyo, Japan) equipped with an ion exchange resin column and an absorbance of 570 nm and 440 nm.

### 2.6. Metabolomics Analysis of Beef Samples

Sample preparation and metabolite extraction. A 100 mg sample was mixed with 200 μL of pre-cooled water and 800 μL of methanol–acetonitrile (1:1, *v*/*v*) in an EP tube. The mixture was transferred to a centrifuge tube, incubated at −20 °C for 1 h, and centrifuged (16,000× *g*) at 4 °C for 20 min. The supernatant was then taken and dried by a high-speed vacuum concentration centrifuge (TGL-16MS, Luxiangyi Centrifuge Instrument Co., Ltd., Shanghai, China). Then, 100 μL of acetonitrile–water (1:1, *v*/*v*) was added for reconstitution, and the supernatant was centrifuged (14,000× *g*) at 4 °C for 15 min for mass spectrometer analysis.

UHPLC-MS/MS of metabolites. The separation of supernatant was performed on an ACQUITY UPLC BEH Amide column (2.1 mm× 100 mm, 1.7 µm) at 25 °C. The gradient elution of mobile phase A (mobile phase A: water + 25 mM ammonium acetate + 25 mM ammonia) and mobile phase B (acetonitrile) was as follows: 0–0.5 min, 95% B; 0.5–7.0 min, 95–65% B; 7.0–9.0 min, 65–40% B; 9.0–10.0 min, 40% B; 10.0–11.1 min, 40–95% B; and 11.1–16.0 min, 95% B. The flow rate and the injection volume were 0.3 mL/min and 5 μL, respectively. The ion source temperature, spray gas pressure, auxiliary heating gas pressure, and curtain gas (CUR) pressure were 600 °C, 60 psi, 60 psi, and 30 psi, respectively. For the TOF MS and product ion scan analysis, the mass ranges were set at *m*/*z* 60–1200 and 25–1200, respectively. The MS/MS detection was performed in information-dependent acquisition (IDA) modes. The IDA parameters of the samples are depicted in Table S3. The raw data collected by UHPLC-MS/MS were pre-processed, identified, and processed with the software SIMCA-P (version 14.1).

### 2.7. Transcriptome Data Processing and Analysis

Extraction and Purification of RNA. Total RNA was extracted from the samples using TRIzol^®^ Reagent following the manufacturer’s protocol (Invitrogen, Carlsbad, CA, USA). The purity and integrity of the extracted RNA were determined by using a NanoPhotometer^®^ spectrophotometer (IMPLEN, Munich, Germany) and a Bioanalyzer 2100 system (Agilent Technologies, Santa Clara, CA, USA). Only total RNA samples with RNA integrity number (RIN) > 8.0 and OD260/OD280 ratios > 2.0 were sequenced.

Transcriptome data analysis. The analysis procedure included cDNA library construction, Illumina platform sequencing, quality control, alignment with the reference genome, and assembling transcripts. The reads were projected onto the yak reference genome by hisat2, and the majority of reads were mapped successfully, with a mapping rate of over 70%. StringTie was used to assemble the transcripts and normalize the fragments per kilobase million (FPKM), which is the basis for selecting differentially expressed genes.

### 2.8. Statistical Analysis

All statistical analyses were performed using SAS 9.12 software (SAS Institute Inc., Cary, NC, USA). R software (University of Auckland, Auckland, New Zealand) was used to plot the graphs. F-ratio tests for variables were conducted. The predicted means and standard errors were generated from the tests, and the significance level was set at <0.05. The tests and visualization were conducted by R software (version 4.1.0). Principal component analysis (PCA) was conducted using SIMCA 14.1 software (Umetrics, Sweden, 2015). Significantly differential metabolites (SDMs) were selected based on the variable importance in projection (VIP) and student’s *t*-test *p*-values. Metabolites with VIP > 1.0 and *p* < 0.05 were considered as SDMs. DEGs (differential genes) were selected based on the logarithmic value of fold change (FC) and *p*-adj calculated by the Benjamini–Hochberg false discovery rate (FDR), and Bonferroni correction was used to account for multiple testing. The genes with |log2 FC| > 1 and *p*-adj < 0.05 were considered DEGs.

## 3. Results and Discussion

### 3.1. Changes in Meat Quality, Amino Acid, and Fatty Acid Profiles

**Figure 1:**
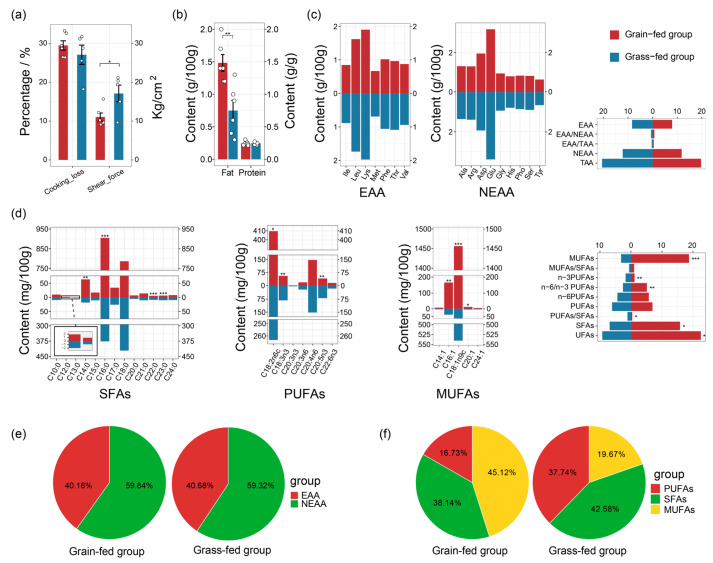
Quality and nutrients of yak meat. (**a**) The cooking loss and shear force. (**b**) The fat and protein content. (**c**) The content and ratio of amino acids. (**d**) The content and ratio of fatty acids. (**e**) The percentage content of amino acids in the yak LL in the grain-fed and grass-fed groups. (**f**) The percentage content of fatty acids in the yak LL in the grain-fed and grass-fed groups. Note: Ile = isoleucine, Leu = leucine, Lys = lysine, Met = methionine, Phe = phenylalanine, Thr = threonine, Val = valine, Ala = alanine, Arg = arginine, Asp = aspartic acid, Glu = glutamic acid, Gly = glycine, His = histidine, Pho = proline, Ser = serine, and Tyr = tyrosine. C10:0 = capric acid, C12:0 = lauric acid, C13:0 = tridecanoic acid, C14:0 = myristic acid, C15:0 = pentadecanoic acid, C16:0 = palmitic acid, C17:0 = margaric acid, C18:0 = stearic acid, C20:0 = arachidic acid, C21:0 = heneicosanoic acid, C22:0 = behenic acid, C23:0 = tricosanoic acid, C24:0 = lignoceric acid, C18:2n6c = linoleic acid, C18:3n3 = γ-linolenic acid, C20:3n3 = eicosatrienoic acid, C20:3n6 = docosatrienoic acid, C20:4n6 = arachidonic acid, C20:5n3 = eicosapentaenoic acid (EPA), C22:6n3 = docosahexaenoic acid (DHA), C14:1 = myristoleic acid, C16:1 = palmitoleic acid, C18:1n9c = oleic acid, C20:1 = gadoleic acid, and C24:1 = nervonic acid. EAA = essential amino acid, NEAA = non-essential amino acid, and TAA = total amino acid. SFAs = saturated fatty acids, MUFAs = monounsaturated fatty acids, PUFAs = polyunsaturated fatty acids, and UFAs = unsaturated fatty acids. Note: * means *p* < 0.05, ** means *p* < 0.01, *** means *p* < 0.001.

The quality of animal meat is closely related to the way it is raised. Meat tenderness is highly affected by the background toughness of animals. The change in background toughness depends on the connective tissue component of muscle, especially the organization of the perimysium [4]. These muscle components will ultimately be influenced by the structure and content of fat and amino acids in beef. Feeding management and nutritional levels, as important environmental factors, directly affect the degree of intramuscular fat deposition and the chemical composition of muscles by regulating the overall growth and energy metabolism of animals. Tenderness and water-holding capacity are fundamental sensory and technological properties of meat that directly determine its palatability, juiciness, and processing yield, thereby constituting core aspects of overall meat quality [4,5]. In this study, shear force was significantly lower in the grain-fed group (indicating improved tenderness), while cooking loss showed no significant difference (Figure 1a).

Nutrients are important indicators of the quality of meat. The protein, amino acid profiles, fat, and fatty acid profiles of the yak LL are shown in Figure 1. The protein content in both groups was approximately 24% (Figure 1b), consistent with a previous study [12]. There was no significant difference in the content of protein and the amino acid profiles determined in the yak LL between the two groups (Figure 1b,c), suggesting that grain feeding did not affect protein and amino acid profiles (*p* > 0.05). Shi et al. [13] found that there was no significant difference in the efficiency of microbial protein synthesis in the rumen of yaks at different dietary levels, and the reason that yaks have maintained the level of protein by conserving N by N excretion and renal N reabsorption may explain this phenomenon [13]. Therefore, this means the yak amino acid content and composition are less dependent on diet.

The intramuscular fat (IMF) content was significantly higher in the grain-fed group (1.48 ± 0.31%) than in the grass-fed group (0.75 ± 0.38%) (*p* < 0.01; Figure 1b), likely contributing to the improved tenderness [14]. Yaks can obtain more energy supplies when supplemented with grain. These nutrients are transported through the bloodstream or lymphatic system to reach peripheral cells and be transformed to fatty acids or TAG for energy supply or storage [14]. However, when explaining this significant difference, the observed differences in animal behavior must be taken into account. The decrease in the amount of grain-fed activity indicates a significant reduction in their daily energy expenditure. Therefore, the increase in intramuscular fat deposition is not simply due to an absolute increase in energy intake but more likely due to a significant positive shift in energy balance. Consistent with this finding, supplemental feeding with alfalfa leaf can alter meat quality [8]. This explains why grain-fed yaks’ fat contents were higher than grass-fed yaks’ fat contents [15]. In Figure 1d, the content of C14:0, C16:0, saturated fatty acids (SFAs), C16:1, C18:1n9c, C20:1, monounsaturated fatty acids (MUFAs), C18:2n6c, and unsaturated fatty acids (UFAs) in the grain-fed group increased, and the ratio of n-6/n-3 polyunsaturated fatty acids (PUFAs) increased to 5.13 (*p* < 0.05). However, the contents of C22:0, C23:0, C18: n3, C18:3n3, C20:5n3, C22:6n3, and n-3 PUFAs in the grain-fed group were lower than the grass-fed group (*p* < 0.05), and the ratio of PUFAs/SFAs also decreased (*p* < 0.05). The PUFAs/SFAs in the two groups were higher than 0.4. As for the percentage of fatty acids, the percentage of SFAs and MUFAs in the grain-fed group was higher than in the grass-fed group (Figure 1f). It is recommended that the intake of SFAs should be reduced and n-3 PUFAs should be increased, which suggests that grain feeding may deteriorate the composition of fatty acids in the yak LL. However, rumen biohydrogenation is also an influencing factor that needs to be considered. It gradually hydrogenates the polyunsaturated fatty acids in the feed, resulting in an increase in the proportion of saturated fatty acids in yak meat. Therefore, grain feeding increases fat content and improves tenderness but may adversely affect the fatty acid profile.

### 3.2. Grain Feeding Affects Flavor Amino Acids and Unsaturated Fatty Acids

**Figure 2:**
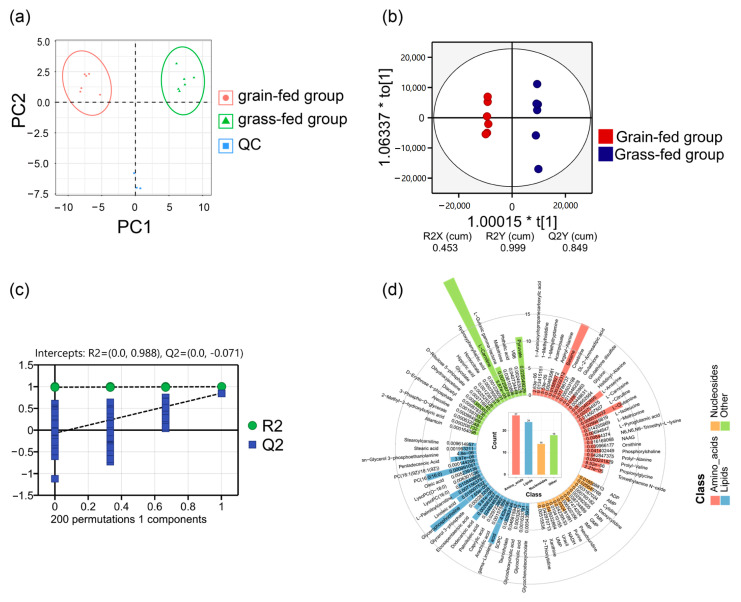
Metabolomics of the grain-fed and grass-fed groups. (**a**) The PCA score plot of metabolites in the yak LL. The red and green circles represent 6 samples of grain-fed group and grass-fed group, respectively. (**b**) The OPLS-DA scores plot of metabolites in the yak LL. (**c**) The permutation test for metabolites in the yak LL. (**d**) The histogram plot of SDMs in the yak LL. Note: NAAG = N-acetylaspartylglutamate, SOPC = 1-stearoyl-2-oleoyl-sn-glycerol 3-phosphocholine, ADP = adenosine 5′-diphosphate, AMP = adenosine monophosphate, FMN = flavin mononucleotide, GMP = guanosine 5′-monophosphate, IMP = inosine 5′-monophosphate, NADH = reduced nicotinamide adenine dinucleotide, UMP = uridine 5′-monophosphate, and VB6 = pyridoxal.

PCA revealed distinct metabolite profiles between the groups (Figure 2a). The quality control (QC) samples, which were prepared by mixing all the experimental samples in equal amounts, clustered tightly and indicated analytical stability. However, the grain-fed and grass-fed samples separated clearly, reflecting dietary influences. The OPLS-DA model was also used to analyze metabolites. The R2Y(cum) = 0.999 and Q2Y(cum) = 0.849 indicated that the explanation and prediction of the model were good (Figure 2b), and the permutation test proved there was no over-fitting in the original model (Q2Y = (0, −0.071)) (Figure 2c).

**Figure 3:**
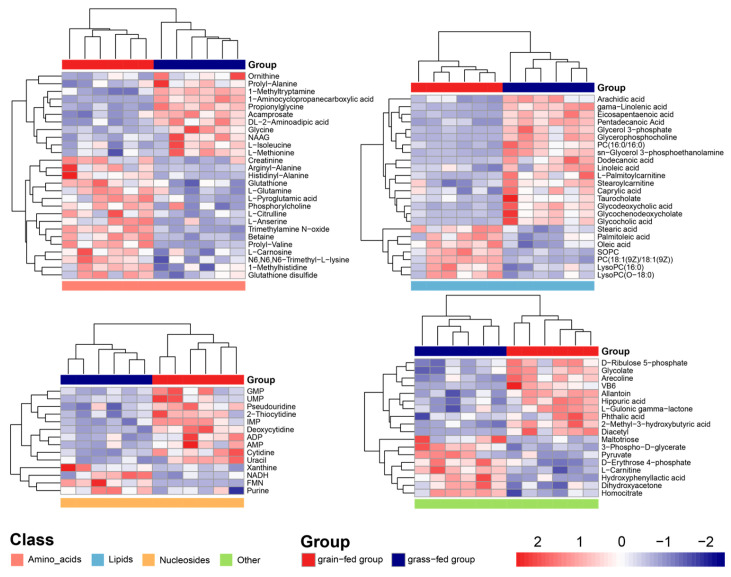
The heatmap of differential metabolites in the yak LL in the grain-fed and grass-fed groups.

Based on the results of OPLS-DA analysis, the metabolites with VIP > 1 and *p* < 0.05 were used as the criterion for screening differential metabolites of the yak LL in the grain-fed and grass-fed groups, and a total of 83 differential metabolites were obtained (Figure 2d), whereas the differential metabolites were composed of 27 amino acid compounds, 24 fatty acid compounds, 14 nucleic acid compounds, and 16 other compounds. The changes of 83 differential metabolites are shown in Figure 3, where 42 and 41 metabolites were upregulated and downregulated in the grain-fed group, respectively. Among the amino acids, L-isoleucine, L-methionine, and glycine were significantly decreased in the grain-fed group. As for lipids, grain feeding essentially caused PUFAs, such as γ-linolenic acid and eicosapentaenoic acid, to exhibit a decreasing trend, but SFAs (stearic acid) and MUFAs (palmitoleic acid, oleic acid) to exhibit an increasing trend. For instance, free amino acids, which can contribute to taste and flavor, are called flavor amino acids, such as bitter amino acids, including isoleucine, leucine, valine, and methionine; sweet amino acids, including alanine, glycine, proline, and serine; and umami amino acids, including glutamic acid and aspartic acid [16]. γ-Linolenic acid and eicosapentaenoic acid (EPA) are indispensable n-3 PUFAs for humans. Therefore, the metabolome results showed that grain feeding enhances the level of flavor nucleosides and decreases the level of bitter amino acids, suggesting the possibility of fattening yaks to obtain better aroma and flavor in the deep-processing process in the future; however, the declines in n-3 PUFAs need to be taken seriously.

**Figure 4:**
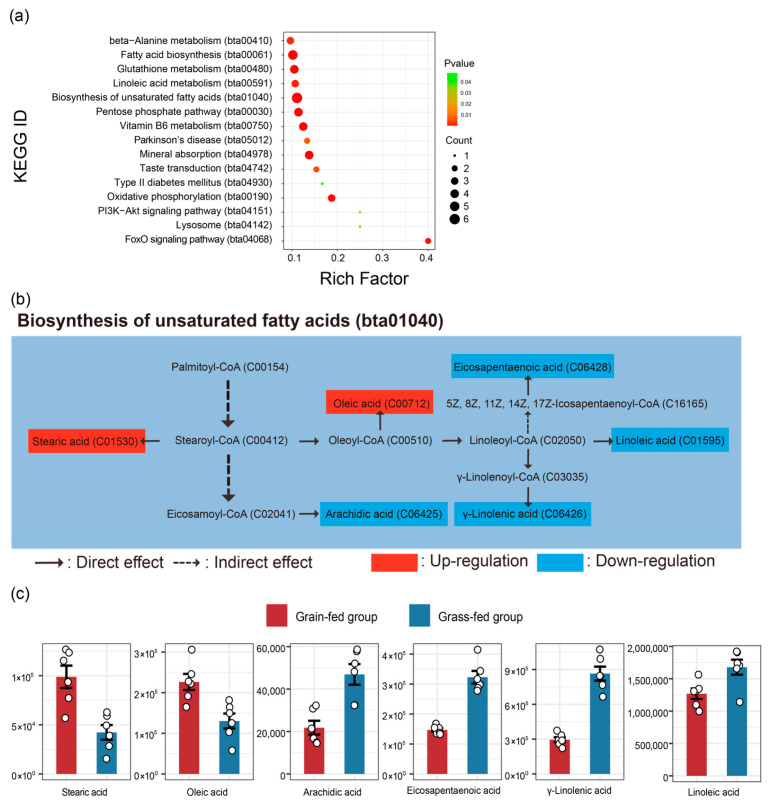
Transcriptomics of the grain-fed and grass-fed groups. (**a**) The bubble plot of the KEGG pathway enrichment analysis of differential genes in the yak LL in the grain-fed and grass-fed groups. (**b**) The pathway for biosynthesis of unsaturated fatty acids. (**c**) The content of differential genes in the yak LL in the grain-fed and grass-fed groups.

Enrichment analysis of differential metabolites revealed a total of 70 metabolites with KEGG id, which were significantly enriched in 47 Kyoto Encyclopedia of Genes and Genomes (KEGG) pathways (*p* < 0.05) (Figure 4a). The KEGG pathways related to fatty acid metabolism, such as fatty acid biosynthesis (bta01040) and linoleic acid metabolism (bta00591), where biosynthesis of unsaturated fatty acids (bta02040) was the richest (Figure 4b). Among the biosynthesis of unsaturated fatty acids, stearic acid and oleic acid exhibited a dramatic increase, whereas others showed a decrease (Figure 4c). Unlike other SFAs, stearic acid has little impact on increasing cholesterol or other blood clot formation [17]. Oleic acid is one of the important MUFAs, and oxidation of oleic acid produces ketones and alcohols, which can improve the flavor of meat. Linoleic acid is an n-6 PUFA. γ-linolenic acid and EPA are n-3 PUFAs. N-6 and n-3 PUFAs are indispensable for humans for normal growth and development, cellular functions and signaling, and the immune response [18]. Therefore, grain feeding may result in the deposition rate of SFAs and MUFAs being faster than that of PUFAs and the synthesis rate of short-chain fatty acids being faster than that of long-chain fatty acids (Figure 4b) [3].

### 3.3. Transcriptomics Reveals Grain Feeding Affects Lipid Metabolism Pathways

**Figure 5:**
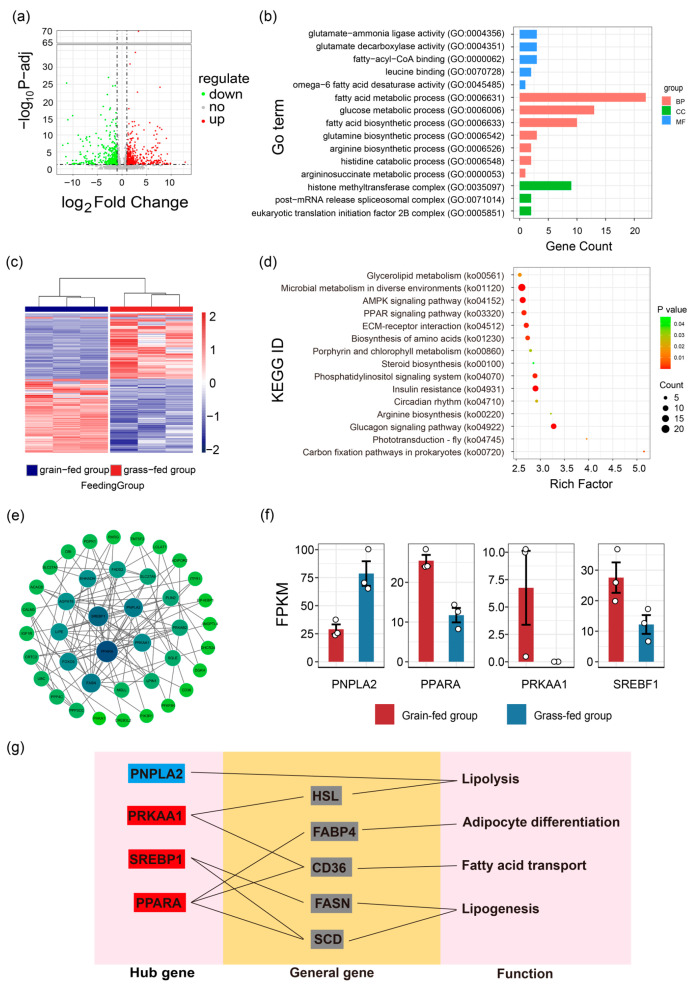
Functional analysis of differential genes and critical genes (**a**) The volcano diagram of genes in the yak LL in the grain-fed and grass-fed groups. (**b**) The histogram of the GO enrichment pathway of differential genes in the yak LL in the grain-fed and grass-fed groups. BP = biological process, CC = cellular component, and MF = molecular function. (**c**) The heatmap of differential genes in the yak LL in the grain-fed and grass-fed groups. (**d**) The bubble plot of the KEGG pathway enrichment analysis of differential genes in the yak LL in the grain-fed and grass-fed groups. (**e**) The network diagram of differential genes related to fatty acid content regulation in the yak LL in the grain-fed and grass-fed groups. (**f**) The histogram plot of FPKM value for Hub genes in the yak LL in the grain-fed and grass-fed groups. (**g**) The relationship between the Hub gene and lipid metabolism. Note: Blue box = down-regulation gene, red box = up-regulation gene, and grey box = general gene.

As shown in the volcano map (Figure 5a), 1047 differential genes were obtained. A total of 570 differential genes were upregulated, while 477 were downregulated in the grain-fed group compared to the grass-fed group. The differential genes were enriched in 44 major KEGG pathways, and the KEGG pathways are shown in Figure 5d. The top five KEGG pathways involving fatty acids included the glucagon signaling pathway (ko04922), steroid biosynthesis (ko00100), PPAR signaling pathway (ko03320), AMP-activated protein kinase (AMPK) signaling pathway (ko04152), and glycerolipid metabolism (ko00561).

The recognized four Hub genes, containing PRKAA1, SREBF1, PPARA, and PNPLA2, encoded the transcriptional regulators related to lipid metabolism (Figure 5f). PRKAA1 is a member of the AMPK family and can indirectly inhibit the activity of HSL, which has been defined as a genetic marker of livestock lipid deposition. PRKAA1 can inhibit lipolysis [19], indirectly promote the expression of CD36, and enhance the biosynthesis of MUFAs [20,21]. The expression of PRKAA1 in muscle can be positively regulated by changing the diet (adding the premix) [22]. In addition, it has obvious tissue specificity. For skeletal muscle, it can specifically affect skeletal muscle development and fat deposition [23]. SREBF1 is regarded as a key gene for lipogenesis, which can regulate the expression of stearoyl-CoA desaturase (SCD) and fatty acid synthase (FASN) [24]. There are two protein products of SREBF1—SREBP1a and -1c. SREBP1a can stimulate lipid synthesis in proliferating cells, while SREBP1c plays an important role in the nutrient regulation of fatty acid and triglyceride (TAG) synthesis in lipogenic organs, such as the liver [25]. It is reported that excessive energy intake activates SREBF1’s expression [26], but SREBP1 is inhibited by n-3 PUFAs (e.g., EPA, DHA) [27]. PPARA is a key member of the PPAR signaling pathway and is involved in the metabolic regulation of fatty acids in the liver, skeletal muscle, and brown fat tissue. PPARA can promote fatty acid transport and lipid metabolism by regulating downstream target genes (SCD, FABP4, CD36) when activated by ligands, such as fatty acids. PNPLA2 is a key gene for the first step of TAG deposition within lipid droplets. Disruption of the PNPLA family can significantly reduce intracellular TAG decomposition and the secretion of TAG-rich lipoproteins [28]. TAG in lipid droplets can be deposited and supply energy when an in vivo baby goes through starvation [29], which is the reason why the expression level of PNPLA2 in the grass-fed group was higher than that of the grain-fed group. Therefore, these Hub genes, which encode transcriptional regulators of lipid biosynthesis, transportation, decomposition, and deposition, may contribute to the phenotypic variation in yak fatness traits (Figure 5g).

### 3.4. The Consistency of Fatty Acid Profiles, Metabolome, and Transcriptome

**Figure 6:**
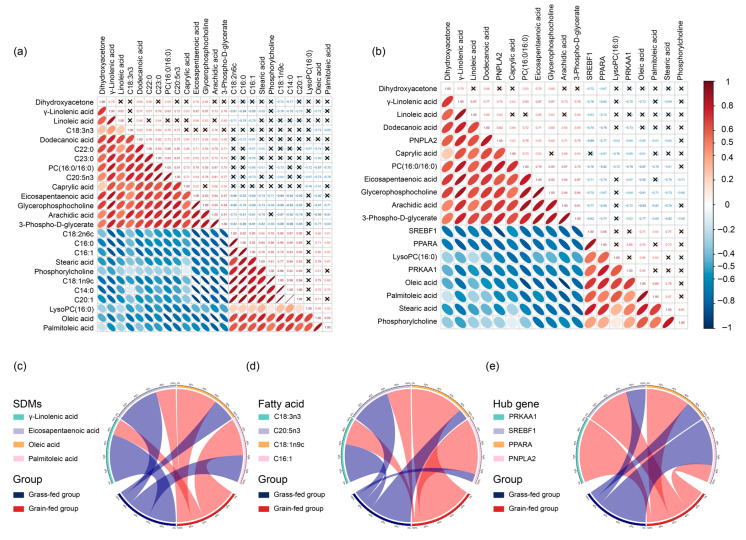
The correlation analysis of fatty acids, metabolites, and genes. (**a**) Metabolites and fatty acids; (**b**) Hub genes and metabolites. The feeding method’s effects on the yak LL by comparing the fluxes in (**c**) differential metabolites, (**d**) fatty acids, and (**e**) Hub genes for the grain-fed and grass-fed groups. Note: ✕ means the correlation ship is not significant.

To assess the consistency between the fatty acid profiles and metabolomic data, as well as the correlations between these profiles and gene expression, Pearson correlation analysis was performed (Figure 6). A strong concordance was observed between the metabolomic and fatty acid profiles (Figure 6a), supporting the reliability of the metabolomic data. Detailed analysis of Hub genes (Figure 6b) revealed specific correlation patterns: PNPLA2 expression showed a significant positive correlation with the levels of γ-linolenic acid, dodecanoic acid, PC (16:0/16:0), arachidic acid, and 3-phospho-D-glycerate. In contrast, SREBF1 expression was negatively correlated with dihydroxyacetone, γ-linolenic acid, dodecanoic acid, PC (16:0/16:0), EPA, glycerophosphocholine, arachidic acid, and 3-phospho-D-glycerate but positively correlated with oleic acid, palmitoleic acid, and stearic acid. Similarly, PPARA expression was negatively correlated with dihydroxyacetone, γ-linolenic acid, linoleic acid, dodecanoic acid, PC (16:0/16:0), EPA, glycerophosphocholine, arachidic acid, and 3-phospho-D-glycerate. PRKAA1 expression was also negatively correlated with γ-linolenic acid, dodecanoic acid, PC (16:0/16:0), and arachidic acid.

The influence of the feeding regimen was further evident in the comparison of metabolic flux or relative abundance between groups. For differential metabolites (Figure 6c), the abundance of γ-linolenic acid and EPA was significantly lower in the grain-fed group, whereas oleic acid and palmitoleic acid showed the opposite trend. A consistent pattern was seen in the fatty acid profiles (Figure 6d). Regarding Hub gene expression (Figure 6e), the levels in the grain-fed group were significantly higher than in the grass-fed group for all except PNPLA2. Collectively, these correlative data suggest that grain feeding is associated with distinct changes in gene expression and metabolic profiles, indicating a potential link between dietary intervention and the observed molecular and biochemical shifts. Further research is needed to confirm the underlying causal mechanisms.

### 3.5. Grain Feeding Improves Fat Deposition for Yak Meat via the PPAR Signaling Pathway

**Figure 7:**
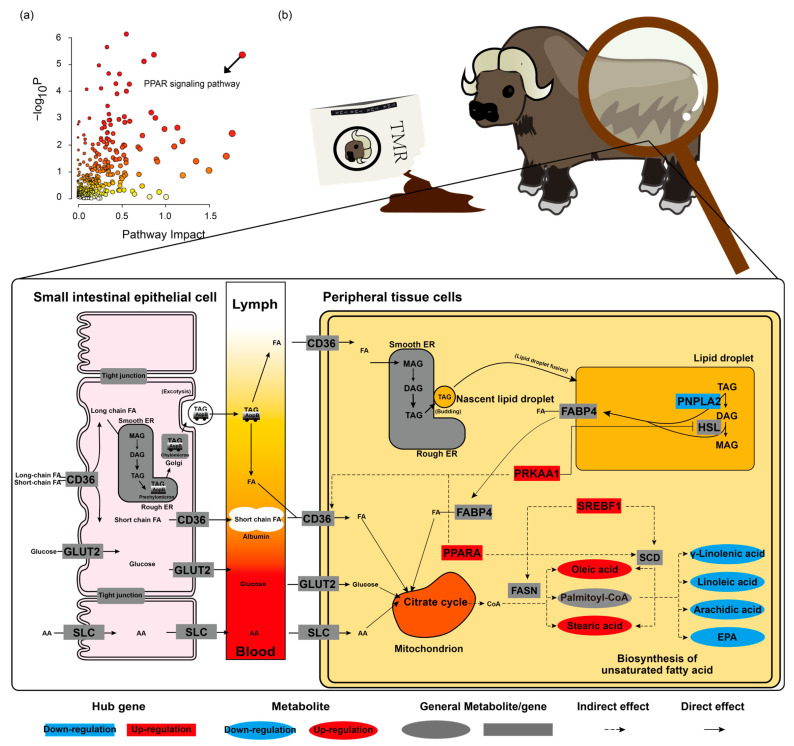
The regulatory mechanisms of grain feeding on yak meat quality, fat, and amino acids. (**a**) The combined analysis of differential metabolites and genes in the grain-fed and grass-fed groups. The bubble color represents the *p*-value, and darker colors represent more significance. (**b**) The regulation of hub genes to metabolism in the yak LL by the combined analysis of the metabolome and transcriptome. Note: TAG = triglyceride, DAG = diglyceride, MAG = monoglyceride, and SCL = solute carrier family 1 (neuronal/epithelial high-affinity glutamate transporter), solute carrier family, including solute carrier family 6 (neurotransmitter transporter), solute carrier family 7 (L-type amino acid transporter), and solute carrier family 15 (oligopeptide transporter).

To gain deeper insights into the impact of grain feeding, we conducted a combined analysis. As shown in Figure 7a, there were 91 significantly enriched pathways (*p* < 0.05), of which the PPAR signaling pathway (pathway impact = 1.8814, −log_10_P = 5.3440) was recognized as the main driving force for the metabolic difference in the yaks in the grain-fed and grass-fed groups (Figure 7b) [30]. The PPAR signaling pathway plays a crucial role in lipid metabolism, and Hub genes are also important genes related to the PPAR signaling pathway. Nutrients from the feed (e.g., short- and long-chain fatty acids, glucose, amino acids) are absorbed by small intestinal epithelial cells. Long-chain fatty acids are re-esterified into triglycerides (TAG) for transport, while others are transported via specific membrane proteins. When yaks take up excess nutrients from TMR, the cells enter an energy-repleted state that activates anabolic programs. The key lipogenic transcription factor SREBF1 is upregulated, which, in turn, enhances the expression of FASN and SCD, crucial enzymes for de novo fatty acid synthesis and desaturation. To facilitate energy storage, yaks increase fatty acid uptake via transporters like CD36 and FABP4 and simultaneously suppress lipid breakdown by inhibiting HSL and reducing PNPLA2 expression. Consequently, triglyceride (TAG) synthesis and deposition are promoted. Within this anabolic state, the deposition of MUFA and SFA synthesized by SCD and FASN occurs more rapidly than that of PUFAs and very long-chain fatty acids, which require additional, rate-limiting elongation and desaturation steps. Therefore, grain feeding promotes fat deposition and increases MUFA and SFA content, but it may even reduce PUFA levels, particularly n-3 PUFAs. In this regard, rumen biohydrogenation is also an important factor that needs to be verified in the future.

While our data strongly suggest that PPAR signaling is a central regulator of the observed metabolic adaptation to grain feeding, several limitations must be considered. The relatively small sample size may limit the statistical power to detect more subtle transcriptional changes and affect the generalizability of our findings. Further, although we controlled for major variables, potential unmeasured confounders, such as individual variation in gut microbiota composition, could influence both lipid absorption and gene expression profiles. Finally, future studies incorporating validation at the protein level, PCR, and functional assays will be crucial to confirm and extend these mechanistic insights.

## 4. Conclusions

This integrated metabolomic and transcriptomic study elucidates the mechanisms by which grain feeding affects yak meat quality. Grain feeding improves tenderness and intramuscular fat deposition. However, it also leads to a deterioration in the fatty acid profile, specifically an increased n-6/n-3 PUFA ratio. Critical genes (*PNPLA2*, *PPARA*, *SREBF1*, and *PRKAA1*) and PPAR signaling manipulated the synthesis of lipids. However, the relatively small sample size and potential unmeasured confounders may affect the generalizability of our findings. Future research should investigate whether dietary strategies, such as supplementing with n-3 PUFA-rich sources like flaxseed oil, can mitigate this nutritional drawback. Also, studies incorporating validation at the protein level and PCR should be considered.

## Data Availability

The data presented in this study are available on request from the corresponding authors. The data are not publicly available due to privacy restrictions.

## Supplementary Materials

The following supporting information can be downloaded at: https://www.mdpi.com/article/10.3390/foods15010172/s1, Table S1: Composition and nutrient levels of TMR; Table S2: The content of fatty acid and amino acid of the TMR; Table S3: The IDA parameters of samples.
