# Supplementary material for: Grain Feeding Improves Yak Meat Tenderness and Lipid Deposition: Meat Quality, Amino Acid and Fatty Acid Profiles, Metabolomics, and Transcriptomics
Source: Foods. 2026 Jan 4;15(1):172. doi: 10.3390/foods15010172 (PMC12785483; doi:10.3390/foods15010172)
Supplement: Supplementary file 1 [file foods-15-00172-s001.zip › foods-4027492-supplementary.pdf]

Table S1 Composition and nutrient levels of TMR.

Ingredient (g/kg)	content	Nutrient level	content
Corn [Grade2, 80%]	740	Integrated net energy (cattle) MC/Kg	1.801
Soybean meal [43%]	35	Beef cattle gain and net energy MC/Kg	1.414
Dried distiller grains with soluble	60	Beef cattle maintain net energy MC/Kg	1.861
Corn fiber	40	Crude protein %	12.205
Distillers dried grains	64	Crude fat %	4.481
Soybean oil	6	Calcium %	0.434
Baking soda	10	Total phosphorus %	0.344
Choline chloride	1	Cattle digestible protein %	6.143
Mountain flour	10	Salt %	1.051
Salt	10	Lysine %	0.362
2% beef premix	20	Dry matter %	6.841
Bacillus	4	Corase fiber %	2.241
Total	1000	Crude ash %	4.627

Note: Each kilogram of the premix contains VA7000-11000 IU, VD3 2600-4000 IU, 1.2-3 mg copper, 6-12 mg iron, 7-15 mg zinc, 44-100 mg manganese, 0.8-2 mg iodine, 0.3-0.6 mg and ≥1.2 mg cobalt. The main raw material composition contains VA, VD_3, VE, nicotinamide (amide compound of VB_3), ferrous sulfate, alkaline copper chloride, zinc sulfate, manganese sulfate, calcium iodate, sodium selenite, cobalt chloride, calcium hydrogen phosphate, sodium chloride, calcium carbonate, propionic acid, rice shell powder (carrier), etc.

Table S2 The content of fatty acid and amino acid of the TMR.

Item	content
Fatty acid [1] (g/100g)	
C16:0	0.54 ± 0.06
C18:0	0.11 ± 0.02
C18:1n9c	0.81 ± 0.10
C18:2n6c	1.83 ± 0.16
C18:3n3	0.35 ± 0.03
Amino acid [2] (g/100g)	
Asp	0.65 ± 0.06
Phe	0.37 ± 0.03
Ala	0.67 ± 0.04
Met	0.12 ± 0.01
Pro	0.83 ± 0.05
Gly	0.35 ± 0.04
Glu	1.65 ± 0.11
Arg	0.30 ± 0.03
Lys	0.30 ± 0.03

Tyr	0.28 ± 0.03
Leu	1.52 ± 0.10
Ser	0.42 ± 0.03
Thr	0.34 ± 0.03
Val	0.46 ± 0.03
Ile	0.21 ± 0.02
His	0.40 ± 0.02

Note: [1] C16:0 = Palmitic acid; C18:0 = Stearic acid; C18:1n9c = Oleic acid; C18:2n6c = Linoleic acid; C18:3n3 = γ-Linolenic acid. [2] Ile = Isoleucine; Leu = Leucine; Lys = Lysine; Met = Methionine; Phe = Phenylalanine; Thr = Threonine; Val = Valine; [2] Ala = Alanine; Arg = Arginine; Asp = Aspartic acid; Glu = Glutamic acid; Gly = Glycine; His = Histidine; Pho = Proline; Ser = Serine; Tyr = Tyrosine.

Table S3 The IDA parameters of samples

analyte	Declustering potential (V)	Collision Energy (eV)	Candidate ions to monitor per cycle	excluded isotope
Yak meat	60<br>-60	30	6	≤4
